# Supplementary material for: Adaptive spectroscopic visible-light optical coherence tomography for clinical retinal oximetry
Source: Commun Med (Lond). 2023 Apr 24;3:57. doi: 10.1038/s43856-023-00288-8 (PMC10126115; doi:10.1038/s43856-023-00288-8)
Supplement: Supplementary file 2 — Supplementary Information-New [file 43856_2023_288_MOESM2_ESM.pdf]

Supplementary Information for

Adaptive spectroscopic visible-light optical coherence tomography for human retinal oximetry

Ian Rubinoff,[1] Roman V. Kuranov,[1,2] Raymond Fang,[1] Zeinab Ghassabi,[3] Yuanbo Wang,[4] Lisa Beckmann,[1] David A. Miller,[1] Gadi Wollstein,[3] Hiroshi Ishikawa,[3,4] Joel S. Schuman,[3] Hao F. Zhang[1,*]

[1.] Department of Biomedical Engineering, Northwestern University, Evanston, IL 60208, USA

[2.] Opticent Inc., Evanston, IL 60201, USA

[3.] Department of Ophthalmology, New York University, New York, NY 10017, USA

[4.] Currently with Department of Ophthalmology, Oregon Health & Science University, Portland, OR 97239, USA

*Corresponding Author, Email: hfzhang@northwestern.edu

Table of Contents

Description	Page(s)
**Supplemental Notes 1*. Ex vivo* phantom validation and *in vivo* comparison**	**3-4**
**Supplemental Notes 2. Statistical advantage of depth averaging**	**4-6**
**Supplemental Notes 3. Influence of Polarization in vis-OCT Retinal Oximetry**	**6**
**Supplemental Notes 4. Longitudinal Chromatic Aberration in vis-OCT Retinal Oximetry**	**7-8**
**Supplemental Notes 5. Accounting for depth-dependent spectra**	**8-9**
**Supplemental Methods 1. Description of adaptive filtering and parameter iterations**	**9-10**
**Figure S1**	**11**
**Figure S2**	**12**
**Figure S3**	**13**
**Figure S4**	**13**
**Figure S5**	**14**
**Figure S6**	**14**
**Table S1**	**15**
**Table S2**	**16**
**Table S3**	**16**
**References**	**17**

Supplementary Notes 1. Ex vivo phantom validation and in vivo comparison

We measured sO_2 values in an *ex vivo* bovine blood phantom using vis-OCT and compared them with a blood-gas analyzer (Rapidlab 248, Siemens Healthcare Diagnostics, Malvern, PA). We pulled a glass capillary tube to an inner diameter of ~ 200 μm (Fig. S1a). We embedded the pulled tube in the middle of a homemade plastic well (Fig. S1b). To reduce specular reflections from the air-glass interface, we added immersion oil (refractive index = 1.52) to the well until the tube was covered by ~ 500 μm of oil.

Next, we prepared oxygenated ($sO_2 \approx$ 100%) and deoxygenated ($sO_2 \approx$ 0%) bovine blood (Quadfive, Ryegate, MT). The hematocrit of blood samples was 45%. To oxygenate blood, we exposed it to a constant stream of pure oxygen while mixing with a magnetic stir bar. We verified that the blood was oxygenated using the blood-gas machine. To deoxygenate blood, we added sodium dithionite to the solution [1]. We monitored the partial pressure of oxygen (pO_2), partial pressure of carbon dioxide (pCO_2), pH, and temperature of the mixture using the blood-gas machine and converted to sO_2 [2]. We continued adding sodium dithionite and measuring sO_2 until the blood was sufficiently deoxygenated. Following oxygenation and deoxygenation, we immediately loaded blood samples into syringes to prevent influences from ambient air.

We used oxygenated and deoxygenated samples to make 17 blood samples between $sO_2 \approx$ 100% and $sO_2 \approx$ 0%. To this end, we mixed oxygenated and deoxygenated samples to create blood of another oxygenation level. We measured sO_2 of the mixed blood using the blood-gas machine. We imaged each blood sample immediately after the blood-gas machine measurement.

Before loading blood into the tube, we flushed the tube with a phosphate-buffered saline (PBS) and heparin solution to prevent clotting or sedimentation. Then, we loaded the blood into a syringe, which was connected to the glass tube by ~ 1 m of plastic tubing. We placed the syringe in a syringe pump (Model-Fusion 100, Chemyx, Inc. Stafford, TX), which flowed the blood at ~ 0.03 mm/s inside the glass tube to prevent clotting or sedimentation.

Before imaging, we focused the beam on the tube by adjusting the tube height and maximizing the intensity of backscattered light. After reaching the best focus, we adjusted the reference arm to place the top of the tube < 100 μm from the zero delay. Then, we imaged the tube using a 512 x 512 raster scan. The optical power incident on the tube was 1.20 mW. After imaging each blood sample, we re-flushed the tube with PBS and heparin solution.

We measured sO_2 with vis-OCT in each blood sample using the Ads-OCT processing proposed in this work. Since scattering factor SSF was not well-agreed upon in the literature, we varied SSF to find the highest spectral fit R^2. We found that the best spectral fit R^2 was reached between $0.02 \leq SSF \leq 0.10$.

We computed 100 vis-OCT sO_2 measurements for each tube. Briefly, we processed and stored all 512 B-scans. Then, we randomly selected and averaged 50 different B-scans from this set for

sO_2 measurement. Then, we refreshed the 512 B-scans and repeated random selection 100 times to reach 100 sO_2 measurements.

Fig. S2a shows tube sO_2 measured by vis-OCT and the blood-gas machine for 17 tubes ranging from $sO_2 \approx 0\%$ to $sO_2 \approx 100\%$. The equation of the best-fit line is $y = (1.01 \pm 0.024)x + 1.28$ and the coefficient of regression is $R^2 = 0.97$. Using the standard error of the slope of the regression fit, we estimate the sensitivity of our measurement in phantoms to be 2.4%. The relationship between the blood-gas machine and vis-OCT sO_2 was nearly a slope of 1 with only ~1% bias. This was within our target accuracy, so we did not apply a post-hoc calibration curve to vis-OCT measurements in this work.

Fig. S2b shows spectral fits (R^2) and best fit SSF for each tube. The average spectral fit R^2 was 0.98, and the average SSF was 0.068. In Fig. S2c, we plot the best-fit SSF *ex vivo* and *in vivo*. In the tubes, we measured SSSF = 0.068 $\pm$ 0.024. For all unique vessels, we measured SSF = 0.064 $\pm$ 0.026, agreeing well with the tube data. Specifically, SSF measurements were 0.061 $\pm$ 0.025, 0.060 $\pm$0.025, 0.062 $\pm$ 0.025, and 0.068 $\pm$ 0.028 for all arteries, large arteries, small arteries, and all veins, respectively. In Fig. S2d, we plot the spectral fit R^2 *ex vivo* and *in vivo*. In tubes, we measured R^2 = 0.98 $\pm$ 0.01. For all unique vessels, we measured average R^2 = 0.95 $\pm$ 0.04. R^2 measurements were 0.95 $\pm$ 0.04, 0.96 $\pm$0.03, 0.93 $\pm$ 0.05, and 0.94 $\pm$ 0.04 for all arteries, major (diameter ≥ 100 μm) arteries, small arteries (diameter < 100 μm), and all veins, respectively. We anticipated that major arteries would have slightly higher R^2 (0.96) than smaller ones (0.93), considering the increased spatial averaging for larger vessels. Nevertheless, R^2 did not have a significant influence on the sO_2 value.

Supplementary Notes 2. Statistical advantage of depth averaging

The depth-resolved slope of NL-SDA-lines (further referred to as the "slope method") was previously used to extract the attenuation coefficient of OCT signals in **Step 8** in Fig. 2 [3-5]. In this work, we found that the depth-resolved average of NL-SDA-lines (further referred to as the "depth-averaging method") was statistically advantageous to the slope method for retinal oximetry. We compared the two methods for sO_2 measurements in the 125 unique human retinal vessels described above. Both sO_2 measurements used identical AdS-vis-OCT processing with identical depth-selection windows. To implement the slope method, only the depth-averaging step, as depicted by Step 8 in the AS-OCT processing, was replaced with a simple linear regression to estimate the slopes of NL-SDA-lines along the z-axis.

Table S3 compares the sO_2 measurement statistics for 125 unique human retinal vessels (72 arteries and 53 veins) for the depth-averaging and slope methods. Success rate indicates the fraction of sO_2 measurements that surpassed Ads-vis-OCT spectral fit R^2 threshold, which is R^2 > 0.80 or R^2 > 0.93 if sO_2 = 100%. For this analysis, we combine sO_2 across all diameters.

For all tested unique arteries, the depth-averaging method had a success rate of 100 %, while the slope method had a success rate of 64 %. For depth-averaging, all arteries had an average sO_2 of 95.1 ± 5.1 %, while the slope method yielded an average of 90.4 ± 8.92 %. The SD of the slope method was 75% higher than that of the averaging method. The lower average spectral fit R^2 for the slope method (0.92), compared with the depth-averaged method (0.95), is consistent with its higher SD. Additionally, we anticipated that the R^2 was still inflated for the slope method since we rejected 36% of vessels based on a low R^2.

For all tested unique veins, the depth-averaging method had a success rate of 100%, while the slope method had a success rate of 66%. For depth-averaging, veins had an average sO_2 of 58.5 ± 4.3 %, while the slope method had an average of 56.8 ± 10.71 %. SD of the slope method was 149% higher than that of the depth-averaging method. Similar to arteries, the slope method had a lower average R^2 (0.92), as compared with the depth-averaging method (0.94). Since we rejected 34 % based on low R^2, we anticipate that the venous R^2 for the slope method was still inflated.

The comparisons between the two methods demonstrate that the depth-averaging method greatly improves the stability of vis-OCT retinal oximetry than the slope method.

Empirically, we found that depth-averaging the natural logarithm of the SDA-lines yielded less noisy spectra, as compared with the slope method (Table S2). We verified these empirical observations by Monte Carlo simulation. To begin, we applied the slope method and depth-averaging method to the equation of a line, which is predicted by Eqn. 7 in **Methods**. (for simplification, removing small effect of LCA):

$$y(x) = -\alpha_1 x + \sigma_N, \tag{S1}$$

where α_1 is an arbitrary constant and σ_N is random, normally distributed noise. x was a 30-pixel vector ranging from 0 to 35 μm for the depth selection window. We used the slope method to compute the slope of Eq. S1 and directly find α_1. We used the depth-averaging method to compute the average value of Eq. S1 (similar to Eq. 8) to find the constant $\beta_1 \propto \alpha_1$. We computed 10^5 iterations of such measurements and then measured their coefficients of variation:

$$CV_{method} = \frac{\sigma_{method}}{|\mu_{method}|}, \tag{S2}$$

where CV_{method} is the coefficient of variation of the measured α_1 or β_1 from each respective method, σ_{method} is the SD of the measured α_1 or β_1 from each respective method, and μ_{method} is the average of the measured α_1 or β_1 from each respective method. We computed CV_{method} for $\alpha_1 = 400$ cm^{-1} to $\alpha_1 = 600$ cm^{-1} in an increment of 20 cm^{-1}, which covered the reported attenuation coefficients of blood in the visible-light spectral range for $W = 0.064$. We used normally distributed noise with amplitude $\sigma_N = 0.02$ (arb. units), which was a relative noise typically observed *in vivo*. For each value of α_1, we calculated $\frac{CV_{\alpha_1}}{CV_{\beta_1}}$, which compared the relative uncertainty of the slope method to the depth-averaging method. For all values of α_1, $\frac{CV_{\alpha_1}}{CV_{\beta_1}}$ converged to 1.67. In general, we found that

value of $\frac{CV_{\alpha_1}}{CV_{\beta_1}}$ was independent of $\alpha_1$and σ_N. This suggested that the depth-averaging method had an intrinsic noise reduction advantage of 67% over the slope method for additive, normally distributed noise.

In reality, however, the SDA-lines follow an exponential decay with the additive noise. We applied a natural logarithm to this function:

$$y_2(x) = \ln\left(e^{-\alpha_1 x} + \sigma_N\right). \tag{S3}$$

One frequent assumption by the slope method is that signal is significantly greater than the noise, or $e^{-\alpha_1 x} \gg \sigma_N$, after which Eq. S3 would converge to a noiseless version of Eq. S1. However, in the human retina, SNR is often low, and this assumption might not be correct. To this end, the noise in Eq. S3 after the natural logarithm is less trivial than in Eq. S1 since there is no longer a linear relationship between α_1 and σ_N. We repeated the simulation described above, except we generated the signal and noise using Eq. S3. Fig. S3 plots $\frac{CV_{\alpha_1}}{CV_{\beta_1}}$ for this simulation. Like the analysis from Eq. S1, the coefficient of variation using the slope method is always higher than that using the depth averaging method. However, the $\frac{CV_{\alpha_1}}{CV_{\beta_1}}$ is not constant and increases with increased α_1. This is because the relationship between α_1 and σ_N is nonlinear in Eq. S3. This has important implications for the slope method in sO_2 calculation since the measured blood spectrum can have different noise levels for different wavelengths and different depth selection windows. In this work, we demonstrated empirically that depth averaging is statistically advantageous over the slope method for sO_2 calculation, consistent with the simulation.

Supplementary Notes 3. Influence of Polarization in vis-OCT Retinal Oximetry

Since the anisotropic form factor and arbitrary orientation of the RBCs change the polarization state of the scattered light unpredictably, the influence of polarization on the measured spectrum needs to be considered. Thus, we mitigated the polarization dependence of the scattered light on the measured spectrum. First, we used a 300 MHz pulse repetition supercontinuum laser, which emits a randomly polarized broadband light changing from pulse to pulse. During the A-scan exposure time of 38 μs of the line-scan camera in our spectrometer, we averaged out multiple random polarization states illuminating RBCs. Second, most photons detected from the blood vessel are multiply scattered photons[6]. As each scattering event alters polarization, returning photons scattered by blood have a broad range of polarizations. As we averaged A-lines at multiple positions across several B-scans, our measured spectrums for blood capture the influence of a wide range of polarizations. Finally, the depth-resolved nature of ADS-vis-OCT and normalization of the signal at the blood maximum eliminates any differences in polarization-dependent backscattering between blood and tissue, which typically influence the relative contribution of out-of-blood tissue scattering and blood absorption in non-depth resolved measurements[7].

Supplementary Notes 4. Longitudinal Chromatic Aberration in vis-OCT Retinal Oximetry

We developed an approach for fitting LCA transfer functions to sO_2 measurement using the physical optics of the human eye. First, we simulated the CFS in the human eye model from Polans et al. [8] using Optic Studio 16 (Zemax, Kirkland, Washington). Since the wavelength ranges and lateral resolutions of the vis-OCT systems used in this study were approximately the same (see **Methods – Vis-OCT Systems**), we used the same CFS for both systems (Fig. S4a). The simulated chromatic focal shift (CFS) CFS is consistent with that previously measured in the human eye [9]. Then, we calculated potential LCA transfer functions using a modified version of the equation used in [10] to account for spectroscopic analysis:

$$A(\lambda, z) = \frac{1}{\left(\frac{z - z_{f_{550}} - CFS(\lambda, z)}{2 z_r(\lambda)}\right)^2 + 1}, \tag{S4}$$

where $z_{f_{550}}$ is the reference focusing depth at 550 nm, $CFS(\lambda, z)$ are the chromatic focal shifts, and $z_r(\lambda)$ are the wavelength-dependent Rayleigh lengths (assumed refractive index = 1.35 and $1/e^2$ spot size = 7 μm). We calculated 41 LCA transfer functions up to $z_{f_{550}}$ = 100 μm above and below the anterior wall of a simulated vessel in $5\ \mu m$ increments. Fig. S4b illustrates a simulated LCA transfer function ($\sqrt{A(\lambda, z)}$) for $z_{f_{550}}$ focused on the anterior vessel wall. Then, we normalized $\sqrt{A(\lambda, z)}$ by its spectral profile at z_n = 12 μm into the simulated vessel lumen, consistent with typical sO_2 measurements, and took its natural logarithm. We found $LCA_{avg}(\lambda, z)$ by averaging the normalized $\ln\left(\sqrt{A(\lambda, z)}\right)$ from z_0 = 16 μm to $z_0 + \Delta z = 46$ μm into the vessel lumen (Eqn. 7), also consistent with typical sO_2 measurements. To create the LCA lookup table, we saved $LCA_{avg}(\lambda, z)$ for each of the 41 focal positions (Fig. S4c).

To understand the potential influence of LCA on sO_2 measurement, we simulated SDA-lines in a vessel consistent with the Beer-Lambert law and the attenuation spectra in Faber et al. [11]. We multiplied $\sqrt{A(\lambda, z)}$ at each focal position with the SDA-lines to account for LCA (Eqn. 1) and took its natural logarithm. We averaged the spectrum at the same depths used to find $LCA_{avg}(\lambda, z)$ (Eqn. 8 in **Methods**). We noted that for all simulated physiological sO_2 measurements (sO_2 = 40% to sO_2 = 100%) and focal positions, the peak-to-peak amplitude of $LCA_{avg}(\lambda, z)$ was less than 0.25 times the peak-to-peak amplitude of $\mu_{t_{blood}}(\lambda)\left[(z_0 - z_n) + \frac{\Delta z}{2}\right]$. We used this relationship to constrain physically reasonable $LCA_{avg}(\lambda, z)$ to avoid overfitting this parameter in the sO_2 measurement. Furthermore, since the above constraint described relative amplitudes only, it was an independent optical power incident on the vessel.

We measured sO_2 in the above simulation without and with LCA fitting described in **Methods**. We measured sO_2 for up to $z_{f_{550}}$ = 100 μm above and below the anterior wall of a simulated vessel in $5\ \mu m$ increments. Measurements were derived from simulated SDA-lines of oxygen-dependent spectra from sO_2 = 40% to 100%. Figs. S4d and S4e plot sO_2 measurements

without fitting the contribution of $LCA_{avg}(\lambda, z)$. Fig. S4d shows sO_2 measurements for $z_{f_{550}}$ = 50 μm above the anterior vessel wall, and Fig. S4e shows sO_2 measurements for $z_{f_{550}}$ = 50 μm below the anterior wall. When $z_{f_{550}}$ = 50 μm above the anterior wall, sO_2 is underestimated. When $z_{f_{550}}$ = 50 μm below the anterior wall, sO_2 is overestimated. Fig. S4f plots sO_2 measurements with fitting the contribution of $LCA_{avg}(\lambda, z)$, as described in **Methods**. For all values of $z_{f_{550}}$, measured sO_2 matches the ground truth sO_2 with a slope of 1.

We recognize this is a simplified approach may not have fully appreciated the exact influence of LCA in each recorded image. Nevertheless, such corrections are based on well-verified aberrations and defocus on the human eye. More precise LCA correction may be reached with a wavefront sensor and adaptive optics to directly measure wavelength-dependent aberrations, although they add expense and complexity to the vis-OCT system. The influence of LCA can also be reduced by employing an achromatizing lens [12] in the sample arm of the system. Additionally, our vis-OCT systems used a focusing beam diameter ($1/e^2$) of 7 μm. Decreasing beam diameter at the cornea and increasing depth of focus can also reduce the influence of LCA on sO_2 calculation, although it may challenge laterally resolving smaller vessels.

Supplementary Notes 5. Accounting for depth-dependent spectra

Fig. S6A shows the spectral stability matrix (SSM) for vessel V1, a vein, in Fig. 3A. The SSM plots how the measured STFT spectrum changes with depth as a function of the selected starting depth z_0 and depth range Δz (see Step 7 in Fig. 2 & Methods – Depth Selection). A lower mean-squared error (MSE) indicates that the measured STFT spectrum has less depth variation. The green box in Fig. 4a highlights the pair of z_0 and Δz where MSE is the lowest, and the black box highlights where it is highest.). In V1, the spectrum is most depth-stable for the longest Δz and least depth-stable for the shortest Δz. Fig. S6B plots measured spectra (normalized between 0 and 1) from the locations of the highest MSEs (black lines) and lowest MSEs (blue lines) in Fig. S6A. There are 9 plots from each of the two regions, each representing nine small shifts in depth (see Methods – Depth Selection). Following the MSE calculation, the spectra plotted in blue change significantly with depth than those plotted in black.

Optimally selected z_0 and Δz varied across the veins investigated in this study. The average selected Δz for veins (n = 53) was 33 μm out of a maximum value of 40 μm. Selected Δz ranged from Δz = 22 μm to Δz = 40 μm.

Fig. S6C shows the SSM for V2 from Fig. 3A. Unlike Fig. S6B, the lowest MSE (green box) is actually at a shorter depth than the highest MSE (black box). Arteries are generally pulsatile and have higher flow velocity than veins, perhaps introducing different flow-dependent SCs at deeper vessel depths. Nevertheless, we show that by finding the z_0 and Δz with the lowest MSE, we

selected the most depth-stable spectrum (Fig. S6B; highest MSE plotted in black, lowest MSE plotted in red).

Like veins, depths with minimized MSE varied across arteries in this study. The average selected Δz in arteries (n = 72) was 33 μm out of a maximum of 40 μm, and selected Δz ranged from Δz = 17 μm to Δz = 40 μm.

Supplementary Methods 1. Adaptive Filtering

Stage 1: We averaged NL-SDA-lines along a 32 μm depth region beyond z_n for each respective B-scan to obtain a 1D STFT spectrum. We calculated sO_2 and spectral fit R^2 from NL-SDA-lines in each B-scan by least-squares fit (see **Methods – Oximetry fitting model**). Then, we applied a threshold of sO_2 > 15% and R^2 > 0.40 and removed B-scans that did not pass. For the smallest analyzed vessels (diameter < 60 μm), we did not perform this step due to increased noises in individual B-scans.

Stage 2: First, we rejected all sO_2 iterations where the spectral fit R^2 < 0.80. We found that noisier arterial spectra resulted in $sO_2 = 100\%$, which saturates at the maximum possible value. Therefore, we also rejected iterations where $sO_2 = 100\%$ and R^2 < 0.93. Next, we sorted the iterations in ascending order of sO_2 and selected the 20 central indexes, acting as a pseudo-median measurement. Among these values, we selected the iteration where the R^2 was the highest. We saved the z_n, W, and S for the selected iteration and accepted the sO_2 with these parameters.

Parameter iterations: We introduced small variations in three parameters we identified as sensitive to sO_2. The first parameter is z_d, the identified depth where the blood signal begins to decay. After STFT, the axial resolution of ~ 9 μm and spatial averaging between B-scans broadens the peak blood backscattering signal, adding uncertainty to its localization. Furthermore, random or unknown parameters, such as speckle noise, erythrocyte spatial distributions, and vis-OCT illumination beam's incidence angle, may contribute to depth-dependent spectroscopic signal differences in vessels. Therefore, we tuned z_d from 6 μm to 14 μm in 8 equidistant steps below the identified peak blood signal and computed sO_2 for each iteration. If a single peak could not be found, perhaps due to noise or spatial averaging, we then tuned z_d from 16 μm to 24 μm in 8 equidistant steps from the peak amplitude of the anterior vessel wall. The second parameter is the scattering scaling factor SSF, which can vary with erythrocyte spatial distributions and multiple scatterings [6]. Based on *ex-vivo* bovine blood sO_2 measurements and human retinal sO_2 measurements, we determined that the strongest regression fits (R^2) were found between SSF $= 0.02$ and SSF $= 0.10$. This is consistent with our previous findings [6]. Therefore, we computed eight iterations of sO_2 for SSF in this range with a step size of 0.01. The third parameter S scales the SDBG amplitude by a small value. Briefly, we measured the SDBG where the vis-OCT signal was attenuated to the noise floor and extrapolated its amplitude at the vessel location by fitting an

exponential curve to the SDBG. Due to the low SNR of the measured spectrum relative to the SDBG, small errors in this extrapolation could alter the measured spectrum. To account for these potential errors, we applied a small correction factor S to the SDBG, $B(\lambda, z)$, before SDBG subtraction from Eq. 1. We tuned S between 0.96 and 1.04 in 9 steps with a step size of 0.01. We measured sO_2 for each iteration (Fig. 2, Step 8). In total, we calculated sO_2 for $8 \times 8 \times 9 = 576$ iterations. We stored measured sO_2 and spectral fit R^2 for each parameter iteration in 3D matrixes.

Fixed-attenuation measurement parameters: We computed sO_2 measurements for a non-adaptive method referred to as 'fixed attenuation' (FA Method; see Results - Comparison with non-adaptive retinal oximetry). Rather than compute optimal depths for normalization and spectroscopic measurement, we used rigid parameters. For z_d, normalization depth, we selected 23 μm below the peak signal from the anterior vessel wall; for z_0, starting depth of spectroscopic measurement, we selected 8 μm after z_d; for Δz, spectroscopic measurement range, we selected 36 μm below z_0. If the rigid parameters did not fit inside of a smaller vessel (diam < 60 μm), we manually reduced the depth range to fit inside the vessel. These parameters were all within the ranges specified in Ads-vis-OCT.

Figure S1: Capillary tube phantom for *ex-vivo* vis-OCT oximetry.

A

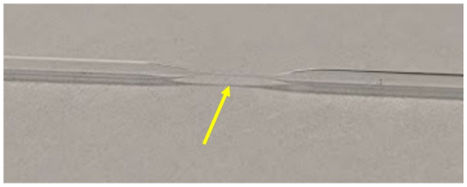

B

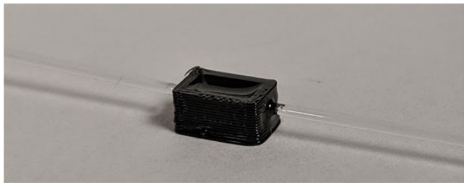

(a) Glass capillary tube pulled to an inner diameter of ~ 200 μm (arrow); (b) Tube inserted in a homemade well under ~ 500 μm of immersion oil.

Figure S2: Results of vis-OCT oximetry in *ex vivo* phantom and comparison to *in vivo* human eye.

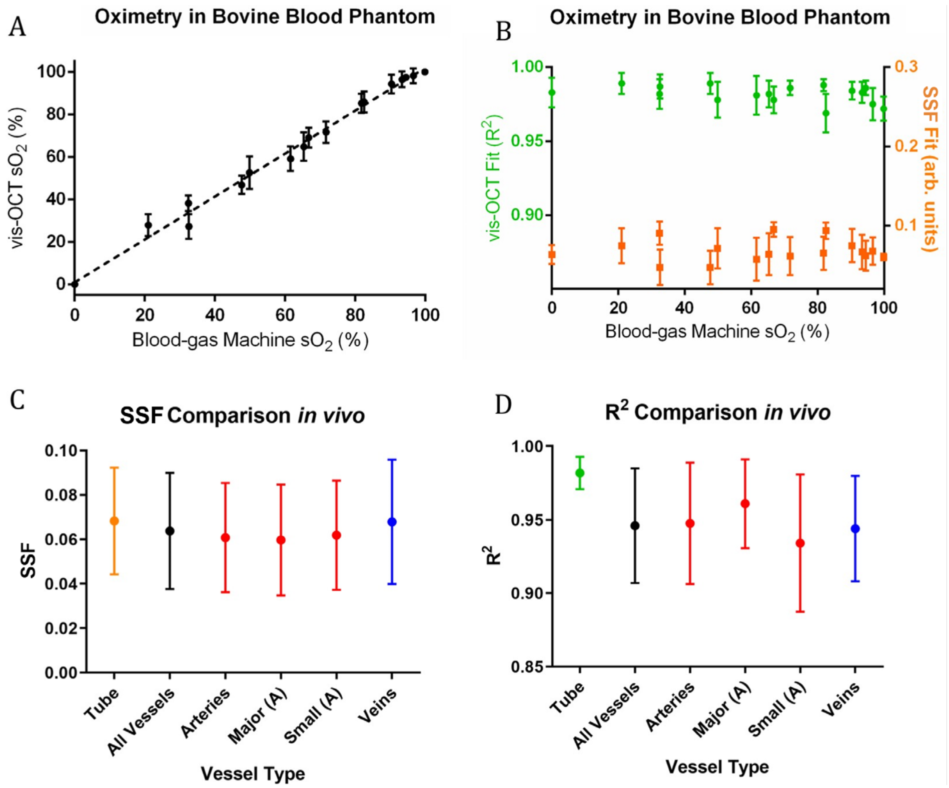

(a) vis-OCT sO_2 measurements in phantom plotted against sO_2 measurements from the blood-gas machine; (b) Distributions of spectral fit R^2 and best fit SSF in phantom (n = 100 for each sO_2 level); (c) Distributions of best fit SSF in phantom compared to the human eye; (d) Distributions of best fit R^2 in phantom compared to human eye. All error bars are standard deviations (n = 1700 for tube, n = 125 for all vessels, n = 72 for arteries, n = 17 for major (A), n = 17 for small (A), n = 53 for veins).

Fig. S3: Comparison of coefficient of variation ratio between slope and depth averaging methods.

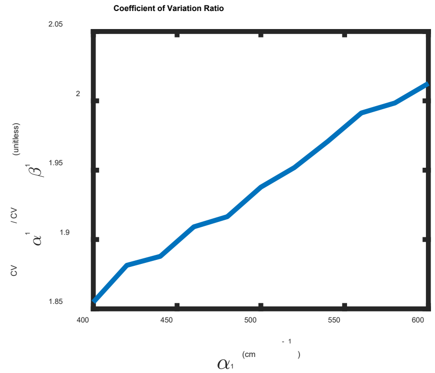

Coefficient of variation ratio between slope and depth averaging methods for estimating attenuation coefficient. Exponential decay model from Eq. S3 is used for calculations. α_1 is attenuation coefficient estimated by slope method; β_1 is proportional to α_1 and estimated by the depth averaging method.

Fig. S4: Simulation of LCA in human eye and influence on vis-OCT retinal oximetry.

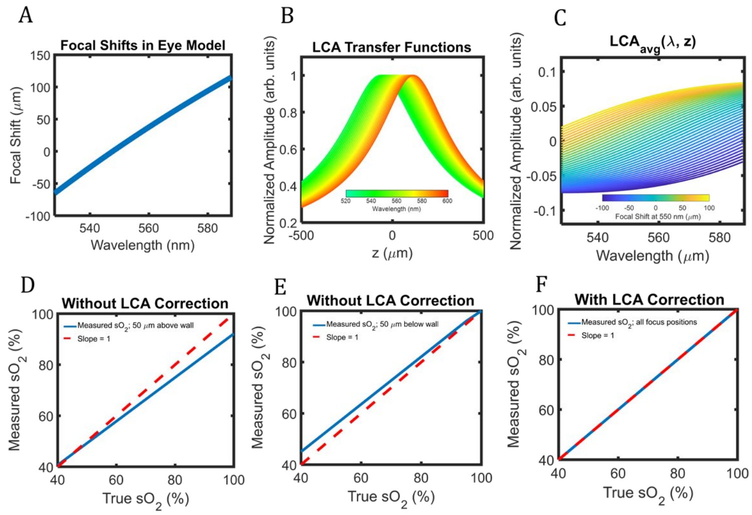

(a) CFS in human eye simulated by Zemax software; (b) Transfer function of the LCA on vis-OCT SDA-lines. Colors depict central wavelength of STFT window; (c) Simulated LCA contribution to measured spectrum after Ads-OCT processing; (d) Simulated sO_2 measurement without LCA correction when focus at 550 nm is 50 μm above the anterior vessel wall; (e) Simulated sO_2 measurement without LCA correction when focus at 550 nm is 50 μm below the anterior vessel wall; (f) Simulated sO_2 measurement with LCA correction for all focus positions

Fig. S5: Example SDA-lines.

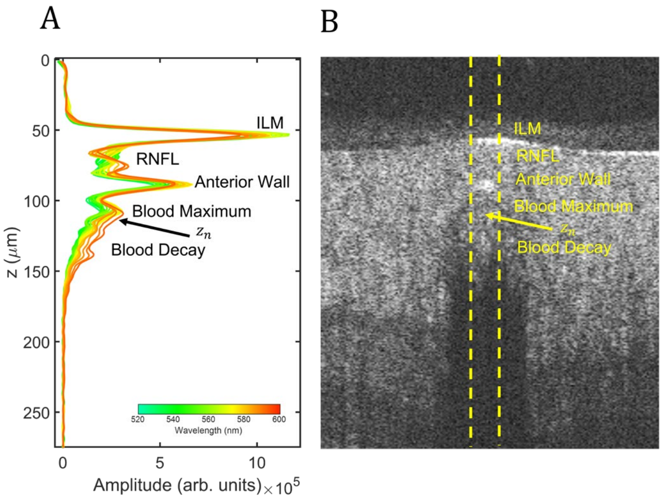

SDA-lines features. (a) SDA-lines from a vein in the human retina (after SDBG correction). The color bar represents the central wavelength of the STFT window. z_d indicates the depth of normalization where SDA-lines start to decay in amplitude; (b) Magnified B-scan where SDA-lines in (a) were measured. SDA-lines were averaged laterally within yellow dashed lines

Fig. S6: Spectral stability analysis in human retinal vessels.

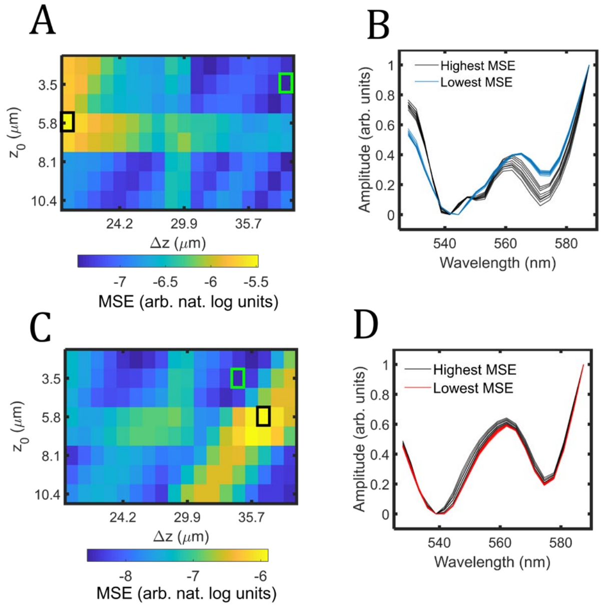

(a) Spectral stability matrix (SSM) for vessel V3 from Fig. S6a. Green box highlights lowest mean-squared-error (MSE) and black box highlights highest MSE; (b) Spectra in V3 after nine depth perturbations for the lowest MSE (blue lines) and highest MSE (black lines) in Fig. S8a, respectively. (c) SSM for vessel V4 from Fig. S6a. Green box highlights MSE and black box highlights highest MSE. (d) Spectra in V4 after nine depth perturbations for the lowest MSE (red lines) and highest MSE (black lines) in Fig. S8c, respectively.

Table S1: Oximetry for each unique vessel.

Unique Vessel	Type	Avg. Diameter (μm)	Avg. sO_2 (%)	Repetitions	Repeatability (SD %)	Avg. Spectral Fit R^2
1	A	138	100.0	3	0	0.972
2	A	134	98.9	2	1.63	0.980
3	A	130	100.0	2	0	0.963
4	A	128	97.1	2	1.06	0.961
5	A	124	99.6	2	0.64	0.965
6	A	122	96.2	3	3.75	0.971
7	A	122	97.8	4	3.81	0.962
8	A	121	99.2	2	1.20	0.946
9	A	121	99.9	3	0.23	0.969
10	A	120	100.0	2	0	0.955
11	A	119	95.5	2	4.88	0.948
12	A	116	99.6	2	0.64	0.953
13	A	116	98.4	2	2.26	0.970
14	A	116	98.6	3	2.48	0.943
15	A	113	97.0	2	4.24	0.966
16	A	105	96.1	2	5.59	0.948
17	A	104	97.9	2	2.97	0.964
18	A	93	92.0	2	2.83	0.966
19	A	89	99.6	2	0.64	0.963
20	A	89	96.2	2	1.27	0.932
21	A	86	91.1	2	2.40	0.950
22	A	86	92.0	3	6.83	0.918
23	A	62	92.9	2	1.13	0.958
24	A	48	91.3	2	2.55	0.912
25	V	174	62.3	2	3.46	0.968
26	V	160	57.0	2	2.12	0.953
27	V	160	58.1	2	0.35	0.954
28	V	157	54.8	3	3.07	0.943
29	V	156	60	3	2.31	0.967
30	V	153	55.4	3	2.97	0.957
31	V	149	55.9	4	4.98	0.951
32	V	146	56.0	2	1.41	0.954
33	V	145	61.0	3	2.06	0.927
34	V	139	58.4	3	3.44	0.918
35	V	135	55.0	2	1.20	0.900
36	V	133	60.1	2	1.27	0.974
37	V	131	54.3	2	0.49	0.940
38	V	128	51.5	2	4.45	0.955
39	V	125	50.6	2	0.78	0.980
40	V	80	59.0	2	2.4	0.977
41	V	70	64.1	2	3.46	0.913
42	V	62	57.7	2	1.41	0.904

All unique vessels analyzed for repeatability. 'A' indicates artery and 'V' indicates vein.

Table S2: All unique major arteries compared with a pulse oximeter.

Subj.	Avg. Pulse Ox. sO_2 (%)	Avg. vis-OCT sO_2 (%)	Num. Unique Major Arteries Measured by vis-OCT	RMSE Between Pulse Ox. and Unique Artery sO_2 (%)
1	100	100	2	0
2	98	99.6	2	1.6
3	99.7	98.9	3	2.0
4	99	95.2	1	3.8
5	95	95.8	1	0.8
6	98	100	1	2
7	99	100	1	1
8	100	98	2	2.8
9	98	100	2	2
10	100	96	2	4
14	98	97.6	5	2.6
17	99	99	3	1.4
Avg.	98.6	98.3	2.1	2.1

Table S3: Comparison of depth-averaging and slope methods for vis-OCT retinal oximetry

	Depth Avg. (A)	Slope (A)	Depth Avg. (V)	Slope (V)
Success %	100 (n = 72)	64 (n = 46)	100 (n = 53)	66 (n = 35)
Average sO_2 (%)	95.1	90.4	58.5	56.8
SD sO_2 (%)	5.1	8.92	4.3	10.71
Average Fit R^2	0.95	0.92	0.94	0.92

Supplementary References

1 Kuranov, R. V. *et al.* Depth-resolved blood oxygen saturation measurement by dual-wavelength photothermal (DWP) optical coherence tomography. *Biomed Opt Express* **2**, 491-504, doi:Doi 10.1364/Boe.2.000491 (2011).

2 Kelman, G. R. Digital Computer Procedure for Conversion of Pco2 into Blood Co2 Content. *Resp Physiol* **3**, 111-+, doi:Doi 10.1016/0034-5687(67)90028-X (1967).

3 Veenstra, C. *et al.* Quantification of total haemoglobin concentrations in human whole blood by spectroscopic visible-light optical coherence tomography. *Sci Rep-Uk* **9**, doi:ARTN 15115 10.1038/s41598-019-51721-9 (2019).

4 Huang, Y. Y. *et al.* Optical Coherence Tomography Detects Necrotic Regions and Volumetrically Quantifies Multicellular Tumor Spheroids. *Cancer Res* **77**, 6011-6020, doi:10.1158/0008-5472.Can-17-0821 (2017).

5 Chong, S. P., Bernucci, M., Radhakrishnan, H. & Srinivasan, V. J. Structural and functional human retinal imaging with a fiber-based visible light OCT ophthalmoscope. *Biomed Opt Express* **8**, 323-337, doi:10.1364/Boe.8.000323 (2017).

6 Fang, R., Rubinoff, I. & Zhang, H. F. Multiple forward scattering reduces the measured scattering coefficient of blood in visible-light optical coherence tomography. *bioRxiv*, 2022.2003.2020.485063, doi:10.1101/2022.03.20.485063 (2022).

7 Smith, M. H., Denninghoff, K. R., Lompado, A. & Hillman, L. W. Effect of multiple light paths on retinal vessel oximetry. *Appl. Opt.* **39**, 1183-1193, doi:10.1364/AO.39.001183 (2000).

8 Polans, J., Jaeken, B., McNabb, R. P., Artal, P. & Izatt, J. A. Wide-field optical model of the human eye with asymmetrically tilted and decentered lens that reproduces measured ocular aberrations. *Optica* **2**, 124-134, doi:10.1364/Optica.2.000124 (2015).

9 Vinas, M. *et al.* Longitudinal Chromatic Aberration of the human eye in the visible and near infrared from Hartmann-Shack wavefront sensing, double-pass and psychophysics. *Invest Ophth Vis Sci* **55** (2014).

10 Faber, D. J., van der Meer, F. J., Aalders, M. C. G. & van Leeuwen, T. G. Quantitative measurement of attenuation coefficients of weakly scattering media using optical coherence tomography. *Opt Express* **12**, 4353-4365, doi:Doi 10.1364/Opex.12.004353 (2004).

11 Faber, D. J. *et al.* Oxygen saturation-dependent absorption and scattering of blood. *Phys Rev Lett* **93**, doi:ARTN 028102 10.1103/PhysRevLett.93.028102 (2004).

12 Chong, S. P. *et al.* Ultrahigh resolution retinal imaging by visible light OCT with longitudinal achromatization. *Biomed Opt Express* **9**, 1477-1491, doi:10.1364/Boe.9.001477 (2018).
