# Supplementary material for: Adaptive spectroscopic visible-light optical coherence tomography for clinical retinal oximetry
Source: Commun Med (Lond). 2023 Apr 24;3:57. doi: 10.1038/s43856-023-00288-8 (PMC10126115; doi:10.1038/s43856-023-00288-8)
Supplement: Supplementary file 3 — Description of Additional Supplementary Files [file 43856_2023_288_MOESM3_ESM.pdf]

Description of Additional Supplementary Files

File Name: Supplementary Data 1

Description: Data used for figures 3, 4, and 5.
