# Supplementary material for: Adaptive spectroscopic visible-light optical coherence tomography for clinical retinal oximetry
Source: Commun Med (Lond). 2023 Apr 24;3:57. doi: 10.1038/s43856-023-00288-8 (PMC10126115; doi:10.1038/s43856-023-00288-8)
Supplement: Supplementary file 5 — Reporting Summary [file 43856_2023_288_MOESM5_ESM.pdf]

nature portfolio

Corresponding author(s): Hao F Zhang

Last updated by author(s): 02/27/2023

Reporting Summary

Nature Portfolio wishes to improve the reproducibility of the work that we publish. This form provides structure for consistency and transparency in reporting. For further information on Nature Portfolio policies, see our Editorial Policies and the Editorial Policy Checklist.

Statistics

For all statistical analyses, confirm that the following items are present in the figure legend, table legend, main text, or Methods section.

n/a	Confirmed	
☐	☒	The exact sample size (n) for each experimental group/condition, given as a discrete number and unit of measurement
☐	☒	A statement on whether measurements were taken from distinct samples or whether the same sample was measured repeatedly
☐	☒	The statistical test(s) used AND whether they are one- or two-sided<br>*Only common tests should be described solely by name; describe more complex techniques in the Methods section.*
☒	☐	A description of all covariates tested
☒	☐	A description of any assumptions or corrections, such as tests of normality and adjustment for multiple comparisons
☐	☒	A full description of the statistical parameters including central tendency (e.g. means) or other basic estimates (e.g. regression coefficient) AND variation (e.g. standard deviation) or associated estimates of uncertainty (e.g. confidence intervals)
☐	☒	For null hypothesis testing, the test statistic (e.g. F, t, r) with confidence intervals, effect sizes, degrees of freedom and P value noted<br>*Give P values as exact values whenever suitable.*
☒	☐	For Bayesian analysis, information on the choice of priors and Markov chain Monte Carlo settings
☒	☐	For hierarchical and complex designs, identification of the appropriate level for tests and full reporting of outcomes
☒	☐	Estimates of effect sizes (e.g. Cohen's d, Pearson's r), indicating how they were calculated

Our web collection on statistics for biologists contains articles on many of the points above.

Software and code

Policy information about availability of computer code

Data collection	We used custom visible-light optical coherence tomography hardware and software to acquire the data used in this work. Retinal oxygenation data were extracted using customized Matlab code.
Data analysis	We used custom code to analyze oxygen saturation in retinal blood vessels. The step-by-step algorithm is described in great detail in the manuscript.

For manuscripts utilizing custom algorithms or software that are central to the research but not yet described in published literature, software must be made available to editors and reviewers. We strongly encourage code deposition in a community repository (e.g. GitHub). See the Nature Portfolio guidelines for submitting code & software for further information.

Data

Policy information about availability of data

All manuscripts must include a data availability statement. This statement should provide the following information, where applicable:

- Accession codes, unique identifiers, or web links for publicly available datasets
- A description of any restrictions on data availability
- For clinical datasets or third party data, please ensure that the statement adheres to our policy

The datasets generated during and/or analyzed during the current study are available from the corresponding author on reasonable request.

Human research participants

Policy information about studies involving human research participants and Sex and Gender in Research.

Reporting on sex and gender	We have equal gender distribution among our volunteers
Population characteristics	Ages ranging from 21 to 62 years old. The median age is 33.5 years old (average 38.1 years old)
Recruitment	We recruited volunteers without known ocular diseases and known systemic diseases.
Ethics oversight	Research IRBs was approved by New York University and Northwestern University

Note that full information on the approval of the study protocol must also be provided in the manuscript.

Field-specific reporting

Please select the one below that is the best fit for your research. If you are not sure, read the appropriate sections before making your selection.

☒ Life sciences ☐ Behavioural & social sciences ☐ Ecological, evolutionary & environmental sciences

For a reference copy of the document with all sections, see nature.com/documents/nr-reporting-summary-flat.pdf

Life sciences study design

All studies must disclose on these points even when the disclosure is negative.

Sample size	Our dataset includes 125 unique vessels from 18 volunteers. Since we present a novel method for noninvasive measurement of retinal oxygen saturation, potential uncertainties and effect sizes in the data were not known until data was acquired. However, the number of measured arteries (n = 72 total; 36 larger arteries; 36 small arteries) and veins (n = 53) were sufficiently high to differentiate statistically significant differences as detailed in the manuscript well beyond 95% confidence.
Data exclusions	Vessel diameter smaller than 35 micrometers were excluded. Data was excluded according to sufficiently low coefficient of regression (R^2) or physically unreasonable sO2, as described in the manuscript.
Replication	The new adaptive algorithm greatly improved the repeatability of retinal oxygenation measurement. Repeated measurements of individual vessels were made when possible, as described in the manuscript. Repeated measurements were performed only at times comfortable and convenient for the volunteer.
Randomization	Volunteers were randomly selected. We analyzed data from volunteers without any known adverse health conditions affecting vision. Arterial vessels were separated into large and small groups due to the statistically significant influence of diameter on oxygen saturation. We did not separate such groups for veins, since there was no statistically significant influence of diameter on oxygen saturation.
Blinding	All imaged vessels from all volunteers were used without any grouping.

Reporting for specific materials, systems and methods

We require information from authors about some types of materials, experimental systems and methods used in many studies. Here, indicate whether each material, system or method listed is relevant to your study. If you are not sure if a list item applies to your research, read the appropriate section before selecting a response.

Materials & experimental systems

n/a	Involved in the study	
☒	☐	Antibodies
☒	☐	Eukaryotic cell lines
☒	☐	Palaeontology and archaeology
☒	☐	Animals and other organisms
☒	☐	Clinical data
☒	☐	Dual use research of concern

Methods

n/a	Involved in the study	
☒	☐	ChIP-seq
☒	☐	Flow cytometry
☒	☐	MRI-based neuroimaging
